# 

**Published:** 1891-11

**Authors:** 


CONTENTS.

Vol. 38. No. 11.

| | PAGE. |
|---|---|
| Growth | 241 |
| Diseases of the Rich | 242 |
| Good Advice to Girls | 243 |
| Water and Health | 243 |
| Children's Teeth | 244 |
| Stammering | 245 |
| Rules for Dyspeptics | 246 |
| Sunday for the People | 247 |
| Hot Water Cures | 247 |
| For a Burn | 248 |
| Milk Impurities | 248 |
| Primitive Practitioners | 249 |
| Bacteria in Milk | 250 |
| Bugs and Hydrophobia | 251 |
| The Best Bread | 251 |
| Good Tea | 253 |
| Cold Food | 253 |
| How to Make a Poultice | 253 |
| Progress on the Congo | 254 |
| From the Dark Ages | 256 |
| Discovery in the Necropolis of Thebes | 258 |
| A Laugh Producing Plant | 259 |
| Ammonia in the Household | 260 |
| Miscellaneous | 261 |
| Life | 261 |

INDEX TO ADVERTISEMENTS OF HALF A PAGE AND OVER.

| | PAGE. |
|---|---|
| The Golden Way | I |
| Lydia E. Pinkham Med. Co. | II |
| Erie Medical Co | III |
| Bovinine | IV |
| Dr. J. C. Ayer & Co | V |
| Herba Vita | VIII |
| Hall's Journal Premiums | IX |
| Rochester Lamp Co. | X |
| Royal Baking Powder | XI |

